# Cardiac safety results from a phase II, open-label, multicenter, pilot study of two docetaxel-based regimens plus bevacizumab for the adjuvant treatment of subjects with node-positive or high-risk node-negative breast cancer

**DOI:** 10.1186/2193-1801-3-244

**Published:** 2014-05-12

**Authors:** Sara A Hurvitz, Linda D Bosserman, David Chan, Christopher T Hagenstad, Frederick C Kass, Frederick P Smith, Gladys I Rodriguez, Barrett H Childs, Dennis J Slamon

**Affiliations:** University of California, Los Angeles, 10945 Le Conte Avenue, PVUB Suite 3360, Los Angeles, CA 90095 USA; Wilshire Oncology Medical Group, Inc., La Verne, CA USA; Torrance Memorial Hospital, Redondo Beach, CA USA; Suburban Hematology and Oncology, Lawrenceville, GA USA; Cancer Center of Santa Barbara, Santa Barbara, CA USA; Georgetown University School of Medicine, Washington, DC USA; South Texas Oncology and Hematology, P.A., San Antonio, TX USA; sanofi U.S., Bridgewater, NJ USA

**Keywords:** Adverse events, Anthracyclines, Antiangiogenic, Congestive heart failure, Trastuzumab

## Abstract

**Purpose:**

Adding antiangiogenic therapy to standard chemotherapy has improved response rates and progression-free survival in metastatic breast cancer (BC) patients. This phase II study evaluated cardiac safety of bevacizumab with/without trastuzumab with two docetaxel-based regimens in early BC.

**Methods:**

127 women with non-metastatic node-positive or high-risk node-negative BC were enrolled. Women with human epidermal growth factor receptor 2 (HER2)-negative BC (n = 93) received docetaxel/doxorubicin/cyclophosphamide (TAC) + bevacizumab, while women with HER2-positive disease (n = 34) received docetaxel/carboplatin/trastuzumab (TCH) + bevacizumab, every 3 weeks for six cycles. Maintenance therapy with bevacizumab alone or bevacizumab plus trastuzumab, respectively, was given every 3 weeks for 52 weeks. The primary objective was to evaluate cardiac safety, as measured by the incidence of ≥ grade 3 clinical congestive heart failure (CHF); the secondary objective was assessment of safety and toxicity.

**Results:**

At least one cardiac adverse event (AE; CHF, cardiomyopathy, or left ventricular dysfunction) was reported in 26.1% of TAC (n = 92) and 17.6% of TCH subjects (n = 34); there were no cardiac deaths. ≥ Grade 3 clinical CHF was observed in 4.3% in the TAC plus bevacizumab stratum and 0% in the TCH plus bevacizumab stratum. A ≥ grade 3 treatment-emergent AE (any kind) related to study treatment was observed in 59.8% in the TAC with bevacizumab and 52.9% in the TCH plus bevacizumab stratum.

**Conclusion:**

Adding bevacizumab to a docetaxel-based regimen with trastuzumab did not appear to increase cardiotoxicity.

**Trial registration:**

ClinicalTrials.gov Identifier: NCT00446030, registered March 8, 2007.

## Introduction

Breast cancer mortality has declined over the past 2 decades; however, it still remains the most common type of cancer in women, accounting for an estimated 29% of all new cases (Siegel et al. 2014). The 5-year survival rate for women with breast cancer is 99% for those with localized disease and 84% for regional disease, and only 24% in patients with distant disease (Siegel et al. 2014). Several studies in human epidermal growth factor receptor 2 (HER2)-normal metastatic breast cancer have reported that the addition of bevacizumab to chemotherapy improves response rates and progression-free survival compared with chemotherapy alone (Miller et al. 2007; Robert et al. 2009; Brufsky et al. 2011; Pivot et al. 2011). Preclinical evidence also suggests that the combination of monoclonal antibodies that target HER2 and vascular endothelial growth factor (VEGF) may act synergistically in HER2 overexpressing cancers (Sweeney et al. 2001; Pegram et al. 2004).

The present study was primarily initiated to evaluate the cardiac safety of bevacizumab when given in combination with a standard-of-care anthracycline-based treatment—docetaxel, doxorubicin, cyclophosphamide (TAC) (Mackey et al. 2013)—in the adjuvant setting. At the time of study initiation, larger studies, such as Eastern Cooperative Oncology Group (ECOG) E5103 (National Cancer Institute), were being planned to evaluate bevacizumab in adjuvant, HER2-negative breast cancer patients. Combining anti-VEGF therapy with anthracycline-based chemotherapy raises concerns regarding cardiac safety, given the association of doxorubicin with an increased risk of congestive heart failure (CHF), and the tendency of bevacizumab to increase blood pressure and, as a result, cardiac “afterload”. The theoretical concern is that a bevacizumab-associated increase in afterload could unmask clinically occult cardiac toxicity from anthracycline, and effectively increase the rate of clinical cardiac adverse events (AEs). The present study was designed in part to provide initial safety data regarding the combination of an anthracycline with bevacizumab for the larger planned studies.

For HER2-positive breast cancer, the use of adjuvant trastuzumab has been shown to improve disease-free survival (DFS) and overall survival (OS) when added to standard chemotherapy (Romond et al. 2005; Slamon et al. 2011). Preclinical data suggesting that HER2-positive breast cancer is particularly reliant on neoangiogenesis (Davidson et al. 1987; Epstein et al. 2002; Yen et al. 2000; Konecny et al. 2004) led to the initiation of several clinical trials that evaluated the combination of trastuzumab and bevacizumab. One of the first of these was a phase II study that enrolled 50 subjects with HER2-positive metastatic breast cancer which reported an asymptomatic cardiac event rate of 36% with a grade 4 cardiac event in 2.0% of subjects (Hurvitz et al. 2009). At the time of the present study’s initiation, planning was underway for BETH (BEvacizumab and Trastuzumab Adjuvant Therapy in HER2-positive Breast Cancer) (Slamon et al. 2013), a large phase III adjuvant study in which patients with HER2-positive breast cancer were randomly assigned to receive docetaxel, carboplatin, and trastuzumab (TCH) with or without bevacizumab. Given that (1) trastuzumab is associated with a low (0.4%) risk of heart failure when given in combination with docetaxel and carboplatin (Slamon et al. 2011), and (2) it is not known if adding bevacizumab to trastuzumab increases the risk of heart failure, our study included a HER2-positive cohort to gauge the cardiac safety of TCH plus bevacizumab.

## Patients and methods

This was a phase II, parallel-group, open-label, noncomparative, multicenter, pilot study (clinicaltrials.gov: NCT00365365). The primary objective was to evaluate the cardiac safety of bevacizumab with/without trastuzumab, as measured by the incidence of ≥ grade 3 clinical CHF, when administered with two different docetaxel-based combination regimens for the adjuvant treatment of subjects with node-positive or high-risk node-negative breast cancer. The secondary objectives were to evaluate the safety and toxicity of these same treatments. The study was originally designed to also evaluate DFS and OS; however, the study was terminated early and follow-up was shortened from 10 years to 2 years; therefore, DFS and OS were not evaluated (second amendment to the protocol). This study was conducted in accordance with Good Clinical Practice and in compliance with the Helsinki Declaration and all applicable local regulatory requirements. Before the performance of any study-related procedures or assessments, each subject signed an institutional review board (IRB)–approved informed consent form (ICF). IRB approval was granted by UCLA-IRB for subjects enrolled at UCLA and by Western IRB for subjects enrolled at other participating sites.

**Figure 1:**
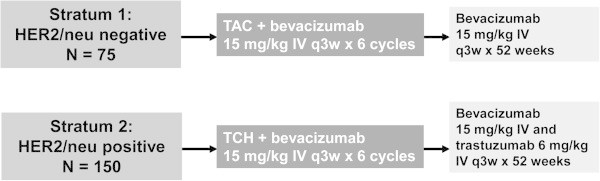
**Study design.** Abbreviations: HER2/neu = human epidermal growth factor receptor-2; TAC = docetaxel, doxorubicin, cyclophosphamide; TCH = docetaxel, carboplatin, trastuzumab.

Subjects were assigned at the time of enrollment to one of two strata: stratum 1 consisted of women with HER2-negative breast cancer who received TAC with bevacizumab while stratum 2 consisted of women with HER2-positive breast cancer who received TCH with bevacizumab (Figure 1). Key inclusion and exclusion criteria are shown in Table 1.

**Table 1:** **Major inclusion and exclusion criteria**

Major inclusion criteria	Major exclusion criteria
● Female 18–70 years old	● History of systemic anticancer therapy for invasive breast cancer, chemotherapy, or radiation therapy
● HER2/neu positive or negative, histologically proven, lymph node-positive or high-risk lymph node-negative breast cancer	● Cardiac disease or poorly controlled hypertension
● Definitive breast surgery within 28 to 60 days consisting of mastectomy; breast conserving surgery with axillary lymph node dissection or sentinel lymph node biopsy for operable breast cancer (T1-3, N0-1, M0)	● Other serious medical issues
● Normal cardiac, hematologic, and liver function	● Minor surgical procedure within 7 days or major surgical procedure within 28 days prior to day 1 of study treatment or any anticipated surgical procedure during the chemotherapy portion of the study

Safety parameters assessed included AEs (nonserious and serious treatment-emergent AEs [TEAEs]) with particular emphasis on cardiac disorders (including grade 3/4 clinical CHF), hematologic disorders, and deaths. A study goal was to categorize CHF by objective criteria rather than using New York Heart Association (NYHA) functional classification, which primarily utilizes physician judgment. Left ventricular ejection fraction (LVEF) was assessed by multigated acquisition scan (MUGA) or echocardiogram (echo). Assessments were performed within 35 days of study enrollment between cycles 3 and 4, as well as 6 and 7; every third cycle during maintenance therapy for trastuzumab/bevacizumab in stratum 2 only; at end of therapy; at post-therapy follow-up/withdrawal; and every 6 months for the 2-year follow-up period. Subjects with LVEF below the institution’s lower limit of normal and a > 10% change from the previous measurement were discontinued from the study. CHF was classified using the National Cancer Institute (NCI) Common Terminology Criteria for Adverse Events (CTCAE) version 3.0. Signs and symptoms of CHF were monitored during the course of trastuzumab therapy as well. Other safety assessments included physical examination, vital signs, and clinical laboratory tests.

It was anticipated that the rate of CHF for bevacizumab would not exceed 2%, while a level of toxicity above 10% would be of great concern. To allow for 5% nonevaluable subjects, the accrual target was 75 subjects per treatment stratum. This was based on a Simon’s 2-stage MiniMax design for a hypothesis test of the null hypothesis of a toxicity-free rate of 90% versus an alternative of 98% with 1-sided significance level of 5% and power of 90%.

## Results

All subjects were women; the majority were Caucasian and under the age of 65 years, with good performance status. Detailed demographic and baseline characteristics for the safety population are presented in Table 2.

**Table 2:** **Demographic and baseline characteristics: safety population**

Treatment	TAC + Bevacizumab (n = 92)	TCH + Bevacizumab (n = 34)	Total (N = 126)
**Gender, female, n (%)**	92 (100.0)	34 (100.0)	126 (100.0)
**Age, years**			
n	92	34	126
Mean	50.7	49.9	50.5
SD	10.09	9.38	9.88
Median	51.5	51.5	51.5
Min, max	28, 70	26, 65	26, 70
**Age group, years, n (%)**			
< 65	84 (91.3)	33 (97.1)	117 (92.9)
≥ 65	8 (8.7)	1 (2.9)	9 (7.1)
**Race, n (%)**			
Caucasian	82 (89.1)	23 (67.6)	105 (83.3)
Black	2 (2.2)	3 (8.8)	5 (4.0)
Asian	2 (2.2)	6 (17.8)	8 (6.3)
Other	6 (6.5)	2 (5.9)	8 (6.3)
**Height, cm**			
n	91	34	125
Mean	164.7	162.3	164.0
SD	7.30	6.99	7.27
Median	165.0	161.0	165.0
Min, max	147, 182	152, 180	147, 182
**Weight, kg**			
n	92	34	126
Mean	76.37	72.09	75.21
SD	17.590	14.766	16.925
Median	72.65	70.20	71.85
Min, max	46.1, 136.8	43.6, 109.5	43.6, 136.8
**Body surface area, m** ^**2**^			
n	91	34	125
Mean	1.826	1.764	1.809
SD	0.2021	0.1716	0.1956
Median	1.810	1.770	1.790
Min, max	1.47, 2.44	1.38, 2.11	1.38, 2.44
**ECOG PS, n (%)**			
0	86 (93.5)	30 (88.2)	116 (92.1)
1	6 (6.5)	4 (11.8)	10 (7.9)
**Surgical procedure, n (%)**			
Lumpectomy	48 (52.2)	25 (73.5)	73 (57.9)
Mastectomy	46 (50.0)	11 (32.4)	57 (45.2)
Other	37 (40.2)	14 (41.2)	51 (40.5)
**Infiltrating ductal carcinoma, n (%)**	76 (82.6)	32 (94.1)	108 (85.7)
**Nuclear grade, n (%)**			
G3	49 (53.3)	20 (58.8)	69 (54.8)
G2	31 (33.7)	14 (41.2)	45 (35.7)
**Disease stage at diagnosis, n (%)**			
IIA	42 (45.7)	12 (35.3)	54 (42.9)
IIB	22 (23.9)	11 (32.4)	33 (26.2)
**TNM staging, n (%)**			
T1N0M0	0	12 (35.3)	12 (9.5)
T1N1M0	20 (21.7)	0	20 (15.9)
T2N0M0	23 (25.0)	8 (23.5)	31 (24.6)
T2N1M0	22 (23.9)	7 (20.6)	29 (23.0)
**Hormone receptor status, n (%)**			
ER+/PR+	47 (51.1)	13 (38.2)	60 (47.6)
ER+/PR-	14 (15.2)	12 (38.2)	26 (20.6)
ER-/PR-	18 (31.5)	7 (20.6)	25 (19.8)
ER-/PR+	2 (2.2)	1 (2.9)	3 (2.4)
**HER2/neu receptor, n (%)**			
Negative	91 (98.9)	0	91 (72.2)
Positive	1 (1.1)	34 (100)	35 (27.8)

*Abbreviations:* ECOG PS = Eastern Cooperative Oncology Group performance status; ER = estrogen receptor; HER2 = human epidermal growth factor receptor; PR = progesterone receptor; SD = standard deviation; TAC = docetaxel, doxorubicin, cyclophosphamide; TCH = docetaxel, carboplatin, trastuzumab; TNM = tumor, node, metastasis.

Among the planned 150 subjects, a total of 127 were enrolled between March and November 2007. At that time, recruitment to the HER2-negative stratum was complete, but enrollment into the HER2-positive stratum was placed on hold while awaiting an amendment that would randomize these subjects into one of two arms—TCH plus bevacizumab (stratum 2 of the current protocol) or an anthracycline-containing arm that was to be used in BETH in some parts of the world. By November 2008, it was determined that this amendment would not be necessary, as there were sufficient safety data already available from the TCH plus bevacizumab stratum of this study, to analyze for purposes of moving forward with the BETH trial and thus the trial was terminated. Overall, a total of 93 subjects in the TAC plus bevacizumab stratum and 34 in the TCH plus bevacizumab stratum were enrolled. One subject in the TAC stratum requested to be discontinued following enrollment but prior to receiving any treatment. Consequently, the safety population consisted of 126 subjects. Among the 93 enrolled subjects in the TAC plus bevacizumab stratum, 52.7% completed study treatment and 67.7% completed follow-up. Among the 46.2% who discontinued treatment, the majority discontinued after cycle 6. The reasons for discontinuation of treatment were AEs (31.2%), subject request (9.7%), poor compliance (1.1%), and “other” (4.3%). Among the 34 enrolled subjects in the TCH plus bevacizumab stratum, 73.5% completed study treatment and 73.5% completed follow-up. A majority of the 26.5% who discontinued from treatment did so after cycle 6. The reasons for discontinuation of treatment were AEs (23.5%) and subject request (2.9%).

### Cardiac toxicity

In the TAC plus bevacizumab stratum (n = 92), at least one cardiac disorder of any grade was reported as an AE in 26.1% of subjects (95% confidence interval [CI], 17.5–36.3) and in the TCH plus bevacizumab stratum (n = 34) in 17.6% of subjects (95% CI, 6.8–34.5). There were no cardiac deaths in either stratum (Table 3). In the TAC plus bevacizumab stratum, 4.3%, and in the TCH plus bevacizumab stratum, 0%, had clinical CHF ≥ grade 3. One subject in the TAC plus bevacizumab stratum experienced two cardiac events, which were clinical CHF and cardiomyopathy. An LVEF reduction of > 10% or an LVEF below the lower limit of normal was seen postbaseline for 23.9% of subjects (95% CI, 15.6–33.9) in the TAC plus bevacizumab stratum and for 23.5% of subjects (95% CI, 10.7–41.2) in the TCH plus bevacizumab stratum (Table 3).

**Table 3:** **Incidence of cardiac adverse events: safety population**

	TAC + Bevacizumab (n = 92)		TCH + Bevacizumab (n = 34)		Total (N = 126)	
	n^a^(%)	95% CI^b^	n^a^(%)	95% CI^b^	n^a^(%)	95% CI^b^
**Subjects with cardiac disorders** ^**c**^	24 (26.1)	(17.5–36.3)	6 (17.6)	(6.8–34.5)	30 (23.8)	(16.7–32.2)
**Subjects with clinical CHF (≥grade 3)** ^**d**^	4 (4.3)	(1.2–10.8)	0	(0.0–10.3)	4 (3.2)	(0.9–7.9)
Cardiac failure congestive	3 (3.3)	(0.7–9.2)	0	(0.0–10.3)	3 (2.4)	(0.5–6.8)
Cardiomyopathy	1 (1.1)	(0.0–5.9)	0	(0.0–10.3)	1 (0.8)	(0.0–4.3)
Left ventricular dysfunction	1 (1.1)	(0.0–5.9)	0	(0.0–10.3)	1 (0.8)	(0.0–4.3)
**Subjects with LVEF reduction > 10% or LVEF < lower limit of normal** ^**e**^	22 (23.9)	(15.6–33.9)	8 (23.5)	(10.7–41.2)	30 (23.8)	(16.7–32.2)
**Cardiac death** ^**f**^	0	(0.0–3.9)	0	(0.0–10.3)	0	(0.0–2.9)

*Abbreviations:* *CI* = confidence interval; *CHF* = congestive heart failure; *LVEF* = left ventricular ejection fraction; *TAC* = docetaxel, doxorubicin, cyclophosphamide; *TCH* = docetaxel, carboplatin, trastuzumab.

^a^Number of subjects who had at least one event at any time during the study.

^b^Exact 2-sided binomial CI.

^c^Includes all subjects with at least one event in the system organ class of Cardiac Disorders.

^d^Includes subjects with at least one ≥ grade 3 event with specified preferred terms.

^e^Includes subjects with LVEF absolute reduction of > 10% from baseline at any time postbaseline, or LVEF < lower limit of normal at any time postbaseline.

^f^Includes subjects with a cardiac disorder event with outcome of death.

### Treatment-emergent adverse events (TEAE)

All subjects experienced at least one TEAE and at least one TEAE was considered related to study treatment (Table 4). At least one ≥ grade 3 TEAE was reported for 67.4% of subjects in the TAC stratum and 61.8% in the TCH stratum (Table 5). A TEAE leading to death occurred in three subjects: two in the TAC stratum (clostridial infection and septic shock) and one in the TCH stratum (ischemic cerebral infarction). The clostridial infection was considered related to docetaxel, bevacizumab, and other study treatment; septic shock was considered related to docetaxel and other treatment; and ischemic cerebral infarction was considered related to docetaxel and bevacizumab. Other bevacizumab-related ≥ grade 3 AEs of Importance in the postsurgical setting include postoperative wound infection in two subjects (2.2%), and wound, impaired healing, and wound dehiscence each in one subject (1.1%). Clostridial infection, neutropenic infection, and perirectal abscess each occurred in one subject (1.1%).

**Table 4:** **Treatment-emergent adverse events (aes) (all grades) reported by ≥20% of subjects in a treatment stratum**

Preferred term, n (%)	TAC + Bevacizumab (n = 92)	TCH + Bevacizumab (n = 34)	Total (N = 126)
Subjects with at least one AE	92 (100.0)	34 (100.0)	126 (100.0)
Fatigue	79 (85.9)	30 (88.2)	109 (86.5)
Nausea	75 (81.5)	28 (82.4)	103 (81.7)
Alopecia	73 (79.3)	28 (82.4)	101 (80.2)
Diarrhea	57 (62.0)	24 (70.6)	81 (64.3)
Arthralgia	47 (51.1)	20 (58.8)	67 (53.2)
Constipation	46 (50.0)	18 (52.9)	64 (50.8)
Epistaxis	43 (46.7)	18 (52.9)	61 (48.4)
Headache	40 (43.5)	17 (50.0)	57 (45.2)
Insomnia	39 (42.4)	16 (47.1)	55 (43.7)
Vomiting	39 (42.4)	12 (35.3)	51 (40.5)
Decreased appetite	38 (41.3)	10 (29.4)	48 (38.1)
Hot flush	33 (35.9)	15 (44.1)	48 (38.1)
Anemia	33 (35.9)	13 (38.2)	46 (36.5)
Lacrimation increased	31 (33.7)	14 (41.2)	45 (35.7)
Neutropenia	39 (42.4)	5 (14.7)	44 (34.9)
Dysgeusia	25 (27.2)	17 (50.0)	42 (33.3)
Stomatitis	30 (32.6)	12 (35.3)	42 (33.3)
Cough	30 (32.6)	8 (23.5)	38 (30.2)
Thrombocytopenia	25 (27.2)	13 (38.2)	38 (30.2)
Dyspepsia	24 (26.1)	13 (38.2)	37 (29.4)
Hypertension	26 (28.3)	11 (32.4)	37 (29.4)
Bone pain	31 (33.7)	5 (14.7)	36 (28.6)
Dyspnea	23 (25.0)	7 (20.6)	30 (23.8)
Oropharyngeal pain	17 (18.5)	10 (29.4)	27 (21.4)
Depression	19 (20.7)	6 (17.6)	25 (19.8)
Leukopenia	19 (20.7)	5 (14.7)	24 (19.0)
Mucosal inflammation	16 (17.4)	7 (20.6)	23 (18.3)
Pain in extremity	20 (21.7)	3 (8.8)	23 (18.3)
Rash	14 (15.2)	9 (26.5)	23 (18.3)
Back pain	14 (15.2)	7 (20.6)	21 (16.7)
Musculoskeletal pain	13 (14.1)	8 (23.5)	21 (16.7)
Edema peripheral	11 (12.0)	7 (20.6)	18 (14.3)
Sinusitis	11 (12.0)	7 (20.6)	18 (14.3)
UTI	10 (10.9)	8 (23.5)	18 (14.3)
Nail disorder	9 (9.8)	8 (23.5)	17 (13.5)
Dry skin	8 (8.7)	8 (23.5)	16 (12.7)
Alanine aminotransferase increased	3 (3.3)	7 (20.6)	10 (7.9)

*Abbreviations:* *TAC* = docetaxel, doxorubicin, cyclophosphamide; *TCH* = docetaxel, carboplatin, trastuzumab; *UTI* = urinary tract infection.

**Table 5:** **Treatment-emergent adverse events (aes) ≥ grade 3, worst grade occurring in at least two subjects overall**

Preferred term, n (%)	TAC + Bevacizumab (n = 92)	TCH + Bevacizumab (n = 34)	Total (N = 126)
Subjects with at least one AE ≥ grade 3	62 (67.4)	21 (61.8)	83 (65.9)
Neutropenia	37 (40.2)	3 (8.8)	40 (31.7)
Leukopenia	16 (17.4)	2 (5.9)	18 (14.3)
Fatigue	6 (6.5)	5 (14.7)	11 (8.7)
Thrombocytopenia	5 (5.4)	5 (14.7)	10 (7.9)
Hypertension	6 (6.5)	4 (11.8)	10 (7.9)
Abdominal pain	4 (4.3)	0	4 (3.2)
Pain	4 (4.3)	0	4 (3.2)
Nausea	3 (3.3)	1 (2.9)	4 (3.2)
Febrile neutropenia	4 (4.3)	0	4 (3.2)
Headache	1 (1.1)	2 (5.9)	3 (2.4)
Dyspnea	3 (3.3)	0	3 (2.4)
Clinical cardiac failure congestive	3 (3.3)	0	3 (2.4)
Diarrhea	1 (1.1)	1 (2.9)	2 (1.6)
Arthralgia	2 (2.2)	0	2 (1.6)
Vomiting	2 (2.2)	0	2 (1.6)
Decreased appetite	1 (1.1)	1 (2.9)	2 (1.6)
Hot flush	1 (1.1)	1 (2.9)	2 (1.6)
Oropharyngeal pain	2 (2.2)	0	2 (1.6)
Anxiety	2 (2.2)	0	2 (1.6)
Neuropathy peripheral	2 (2.2)	0	2 (1.6)
Back pain	0	2 (5.9)	2 (1.6)
Asthenia	1 (1.1)	1 (2.9)	2 (1.6)
Hyperglycemia	1 (1.1)	1 (2.9)	2 (1.6)
Weight decreased	2 (2.2)	0	2 (1.6)
Proteinuria	1 (1.1)	1 (2.9)	2 (1.6)
Neutrophil count decreased	2 (2.2)	0	2 (1.6)
Postoperative wound infection	2 (2.2)	0	2 (1.6)

*Abbreviations:* *TAC* = docetaxel, doxorubicin, cyclophosphamide; *TCH* = docetaxel, carboplatin, trastuzumab.

## Discussion

In the present study, cardiac safety was manageable in both treatment strata, with the number of ≥ grade 3 events below the prespecified threshold of discontinuation for these combinations. Twenty-four subjects (26.1%) treated with TAC plus bevacizumab and six subjects (17.6%) treated with TCH plus bevacizumab experienced a cardiac AE. ≥ Grade 3 clinical CHF was experienced by four subjects (4.3%) treated with TAC plus bevacizumab but no subjects (0%) treated with TCH plus bevacizumab. The incidence of CHF in the TAC stratum in the present study was higher than observed in similar AC-T stratum of the Breast Cancer International Research Group (BCIRG) 006 study (Slamon et al. 2011). Despite using more stringent criteria and review by a blinded, independent cardiac review panel, the rates of CHF in the non-trastuzumab and the non-anthracycline containing strata of BCIRG 006 were 0.7% of those treated with doxorubicin and cyclophosphamide followed by docetaxel and 0.4% with TCH, respectively (Slamon et al. 2011).

In addition, more subjects treated with TCH plus bevacizumab in our study experienced LVEF reduction > 10% compared with subjects enrolled in the TCH-alone arm of BCIRG 006 (23.5% vs. 9.4%, respectively) and more subjects treated with TAC plus bevacizumab in our study experienced LVEF reduction > 10% compared with the AC-T arm of BCIRG 006 (23.9% vs. 11.2%, respectively) (Slamon et al. 2011). However, the higher rate of decrease in LVEF > 10% in our study compared with that in BCIRG 006 might be because BCIRG 006 does not include patients whose LVEF dropped to < 10% but has measurements less than the lower limit of normal, while our study did. In the TAC plus bevacizumab stratum of the current study, 4.3% of subjects experienced CHF. This is higher than rates of 0%–2% observed in other anthracycline-based adjuvant studies (Martin et al. 2003; Henderson et al. 2003; Citron et al. 2003), including rates of 3% in the BCIRG 001 study and 0.2% in the Spanish Breast Cancer Research Group (GEICAM) 9805 study with TAC (Mackey et al. 2013; Martin and Hall 2005). Our rate is also higher than the 1.6% determined from a meta-analysis of 3784 patients with breast cancer treated with bevacizumab (Choueiri et al. 2011). This difference may be a result of the concomitant treatment with an anthracycline in the majority of subjects in our study (73%) compared with 16% of patients in the meta-analysis that likely contributed to the higher rate of CHF in our study (Choueiri et al. 2011). The phase III study AVEREL (Avastin [Bevacizumab] in Combination With Herceptin [Trastuzumab]/Docetaxel in Patients With HER2-Positive Metastatic Breast Cancer) enrolled 424 patients with HER2-positive metastatic breast cancer who were randomly assigned to docetaxel plus trastuzumab with or without bevacizumab. ≥ Grade 3 CHF was noted in 5.1% of patients in the bevacizumab arm compared with 2.9% in the control arm (Gianni et al. 2013).

The small number of subjects in the current study and different assessment methods make cross-trial comparisons of cardiac effects difficult. However, the higher overall rate of CHF, particularly LVEF reduction > 10% or LVEF below the lower limit of normal observed in our study, is a concern. It should be noted that this study was conducted in 2007–2008, prior to the US Food and Drug Administration’s November 2011 decision to revoke approval of the breast cancer indication for bevacizumab based on an assessment of risk-benefit data in women with metastatic breast cancer (United States Food and Drug Administration 2011).

Assessing the incidence of grade 3/4 clinical CHF as a primary end point proved challenging. The present study did not use the NYHA criteria for CHF, but rather the NCI CTCAE criteria as used by Miller et al. in ECOG 2100 (Miller et al. 2007). CTCAE contains a variety of terms that suggest CHF (e.g., dyspnea, shortness of breath), but CHF can present with a wide range of signs/symptoms. Many of the symptoms of CHF overlap with other disease states such as obesity, chronic obstructive pulmonary disease, kidney failure, edema, and liver failure. Using CTCAE, an investigator might simply identify a symptom as dyspnea, shortness of breath, or fatigue and not associate the symptom with left ventricular systolic dysfunction if a recent LVEF determination was not performed.

There are several important limitations to the study. While the safety data in this study allow a gauge of the cardiac safety in the TAC plus bevacizumab and TCH plus bevacizumab arms of the study, it must be kept in mind that the quantity of safety data collected in the present study was less than originally intended, especially for the TCH plus bevacizumab arm. The sample size of the study was determined based on the anticipation that the rate of CHF for bevacizumab would not exceed 2%, while a level of toxicity above 10% would be of great concern. The lower than needed number of subjects enrolled in the TCH arm and decrease in the follow-up period from 10 to 2 years were unanticipated challenges that preclude drawing firm conclusions regarding the relative cardiac safety of TCH plus bevacizumab.

## Conclusion

Cardiac and other toxicities observed in both treatment strata were consistent with those expected from the study treatments administered to subjects with node-positive or high-risk node-negative breast cancer without bevacizumab. The number of ≥ grade 3 cardiac AEs was below the prespecified threshold of discontinuation for these combinations. That said, the overall higher rate of CHF, particularly LVEF reduction > 10% or LVEF below the lower limit of normal, observed in the TAC plus bevacizumab arm is a concern. The safety data available for the TCH plus bevacizumab arm indicated that addition of bevacizumab to a docetaxel regimen with trastuzumab did not increase cardiotoxicity to a threshold supporting cessation of further/future evaluation of this combination. Originally intended secondary end points of DFS and OS were not evaluated due to the reduced follow-up period resulting from a second amendment to the protocol. Because the number of subjects enrolled into the TCH with bevacizumab arm was reduced to half of the originally intended number, all conclusions related to the safety of TCH with bevacizumab should be interpreted with caution.
